# A simple technique for repositioning of the mandible by a surgical guide prepared using a three-dimensional model after segmental mandibulectomy

**DOI:** 10.1186/s40902-017-0113-5

**Published:** 2017-06-25

**Authors:** Akinori Funayama, Taku Kojima, Michiko Yoshizawa, Toshihiko Mikami, Shohei Kanemaru, Kanae Niimi, Yohei Oda, Yusuke Kato, Tadaharu Kobayashi

**Affiliations:** 1 Department of Tissue Regeneration and Reconstruction, Division of Reconstructive Surgery for Oral and Maxillofacial Region, Niigata University Graduate School of Medical and Dental Sciences, 2-5274 Gakkocho-Dori, Cyuo-ku, Niigata, 951-8514 Japan; 2 Department of Oral and Maxillofacial Surgery, Matsumoto Dental University School of Dentistry, Nagano, Japan

**Keywords:** Segmental mandibulectomy, Repositioning of the condylar head, Surgical device, Autopolymer resin, Mandibular reconstruction

## Abstract

**Background:**

Mandibular reconstruction is performed after segmental mandibulectomy, and precise repositioning of the condylar head in the temporomandibular fossa is essential for maintaining preoperative occlusion.

**Methods:**

In cases without involvement of soft tissue around the mandibular bone, the autopolymer resin in a soft state is pressed against the lower border of the mandible and buccal and lingual sides of the 3D model on the excised side. After hardening, it is shaved with a carbide bar to make the proximal and distal parts parallel to the resected surface in order to determine the direction of mandibular resection. On the other hand, in cases that require resection of soft tissue around the mandible such as cases of a malignant tumor, right and left mandibular rami of the 3D model are connected with the autopolymer resin to keep the preoperative position between proximal and distal segments before surgical simulation. The device is made to fit the lower border of the anterior mandible and the posterior border of the mandibular ramus. The device has a U-shaped handle so that adaptation of the device will not interfere with the soft tissue to be removed and has holes to be fixed on the mandible with screws.

**Results:**

We successfully performed the planned accurate segmental mandibulectomy and the precise repositioning of the condylar head by the device.

**Conclusions:**

The present technique and device that we developed proved to be simple and useful for restoring the preoperative condylar head positioning in the temporomandibular fossa and the precise resection of the mandible.

## Background

In many cases of oral carcinoma, especially lower gingival squamous cell carcinoma, and in cases of a primary intraosseous tumor such as ameloblastoma, segmental mandibulectomy is performed depending on the extent of tumor development [1–3]. For precise resection of the tumor, it is important to accurately determine the position and direction for cutting of the mandible since the mandible is a three-dimensionally complex structure. Accurate repositioning of the condylar head in the temporomandibular fossa is required for reconstruction after segmental mandibulectomy in order to maintain the maxillomandibular relationship, though accurate repositioning is often difficult, especially when the proximal segment has no tooth [4]. Various techniques and devices for the fixation of mandibular segments after segmental mandibulectomy have been reported [3, 5–10]. In this report, a simple method using a surgical device made from an autopolymer resin that enables both precise resection of the mandible and restoration of the bone segment position is described.

## Methods

### Case 1

**Fig. 1:**
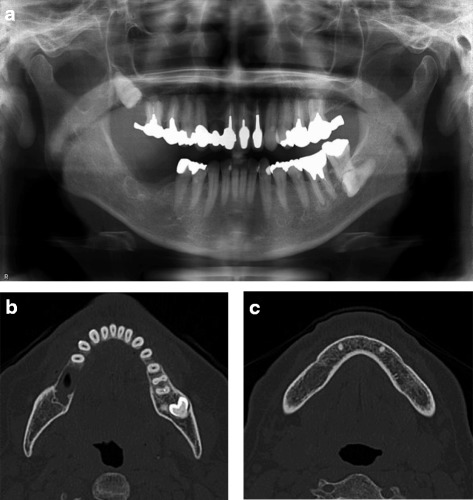
Preoperative **a** panoramic radiograph and **b**, **c** CT image in case 1. Bone resorption was observed in the molar region of the right mandible, and the lesion remained within the mandibular bone

A 57-year-old female was diagnosed with ameloblastoma in the mandible on the right side. A radiolucent lesion of the right mandible was observed on a panoramic radiograph (Fig. 1a) and a computed tomography (CT) image (Fig. 1b, c), and segmental resection of the mandible without the surrounding soft tissue was scheduled.

**Fig. 2:**
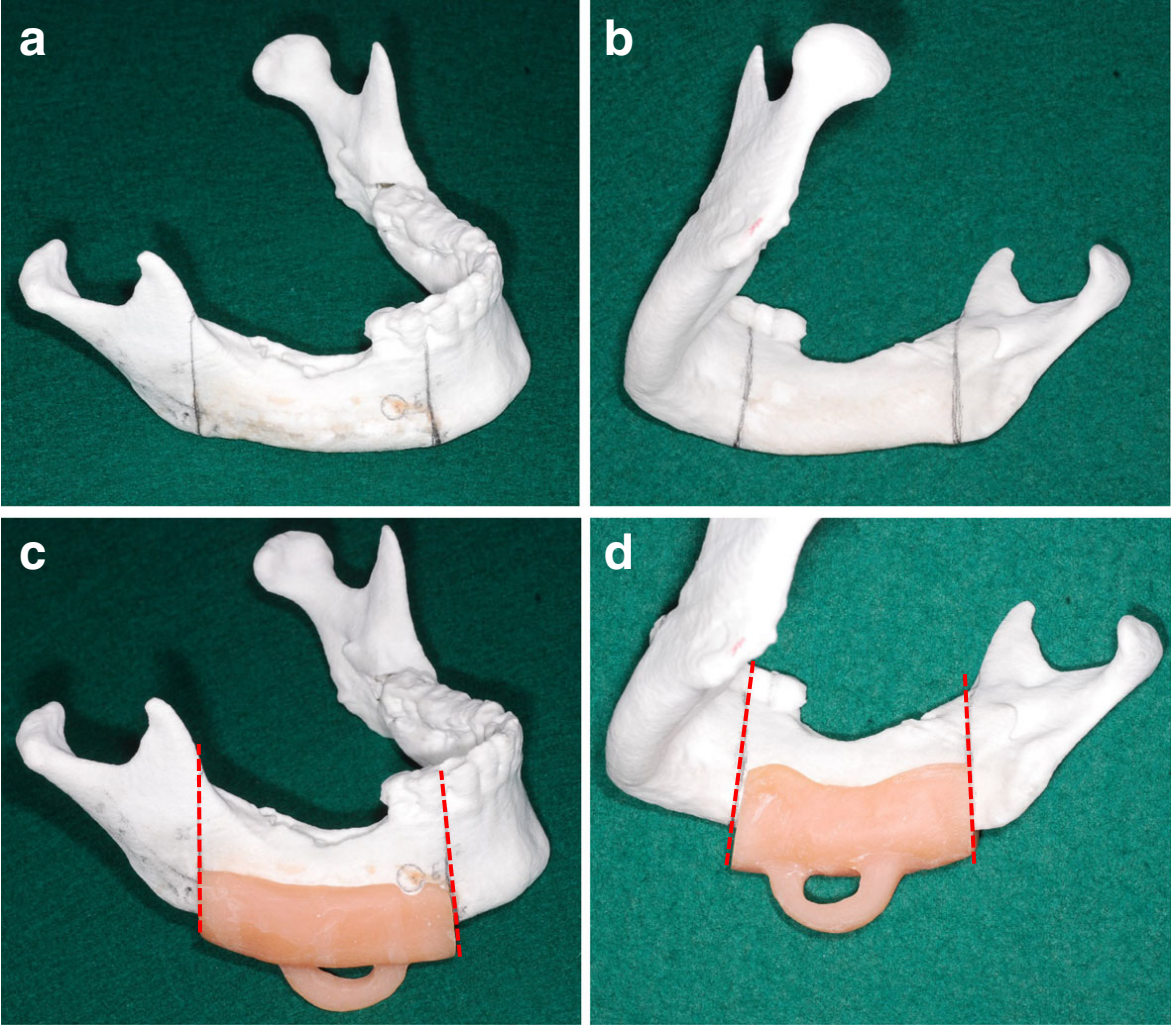
Procedure for making the surgical device in case 1. **a**, **b** Based on the CT image, the range of segmental mandibular resection was marked on the 3D model. **c**, **d** The surgical guide to control the position and direction of osteotomy was made from an autopolymer resin

As the first step, a three-dimensional (3D) model of the mandible was created by a 3D printer using DICOM data of CT. The extent of segmental resection with an approximately 10-mm safety margin was determined, and the resection lines were marked on the 3D model (Fig. 2a, b). Next, a surgical guide made from an autopolymer resin was prepared to control the position and direction of osteotomy. The autopolymer resin in a soft state was pressed against the lower border of the mandible and buccal and lingual sides of the 3D model. After hardening, it was shaved with a carbide bar to make the proximal and distal parts parallel to the resected surface in order to determine the direction of mandibular resection (Fig. 2c, d). Then, a titanium reconstruction plate was bent to conform closely to the form of the 3D model.

**Fig. 3:**
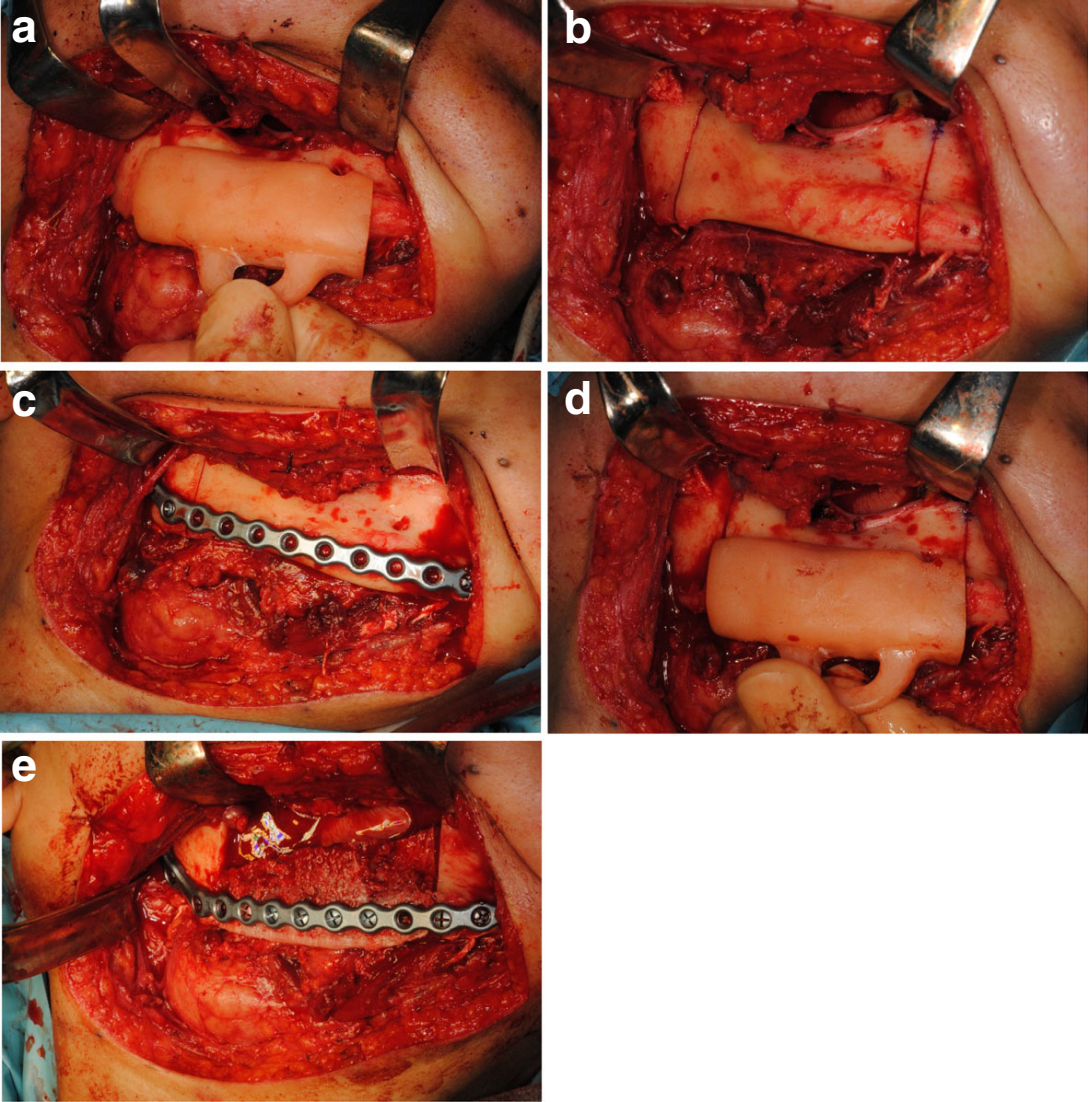
Surgical procedure in case 1. **a** The device was stable on the mandible. **b** Osteotomy was performed only on the buccal cortical bone using a sagittal saw with the device. **c** The pre-bent titanium reconstruction plate was provisionally fixed to the mandible. **d** The device was reinstalled again, and segmental resection of the mandible was completely performed along the proximal and distal edges of the device. **e** The titanium reconstruction plate was fixed at the previous temporarily fixed position, and the free iliac bone was transplanted

**Fig. 4:**
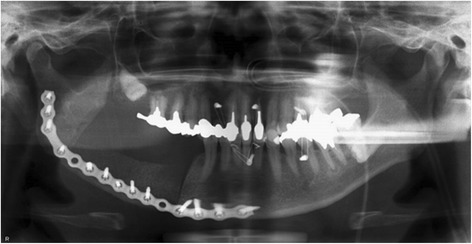
Postoperative panoramic radiograph in case 1. Both the occlusion and position of condylar heads in the temporomandibular fossae were restored to the preoperative position

Segmental mandibular resection was performed via submandibular approach. The periosteum was separated from the bone, and the surgical device was attached (Fig. 3a). The osteotomy was performed only on the proximal and distal buccal cortical bone using a sagittal saw along the proximal and distal edges of the device (Fig. 3b). After that, the pre-bent titanium reconstruction plate was provisionally fixed to the mandible (Fig. 3c). After detaching the titanium reconstruction plate, the device was reinstalled again and segmental resection of the mandible was completely performed (Fig. 3d). The titanium reconstruction plate was fixed at the previous temporarily fixed position, and free iliac bone was transplanted (Fig. 3e). Both the occlusion and position of condylar heads in the temporomandibular fossae could be restored to the preoperative position (Fig. 4).

### Case 2

**Fig. 5:**
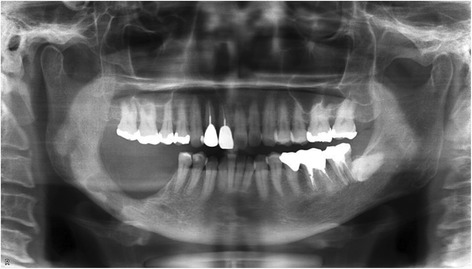
Preoperative panoramic radiograph in case 2. A radiolucent area was observed in the molar region of the right mandible because of recurrent squamous cell carcinoma

The patient was a 61-year-old female who had recurrent lower gingival squamous cell carcinoma in the right side of the mandible (Fig. 5). Segmental resection of the mandible including the surrounding soft tissue with a 10-mm safety margin was scheduled.

**Fig. 6:**
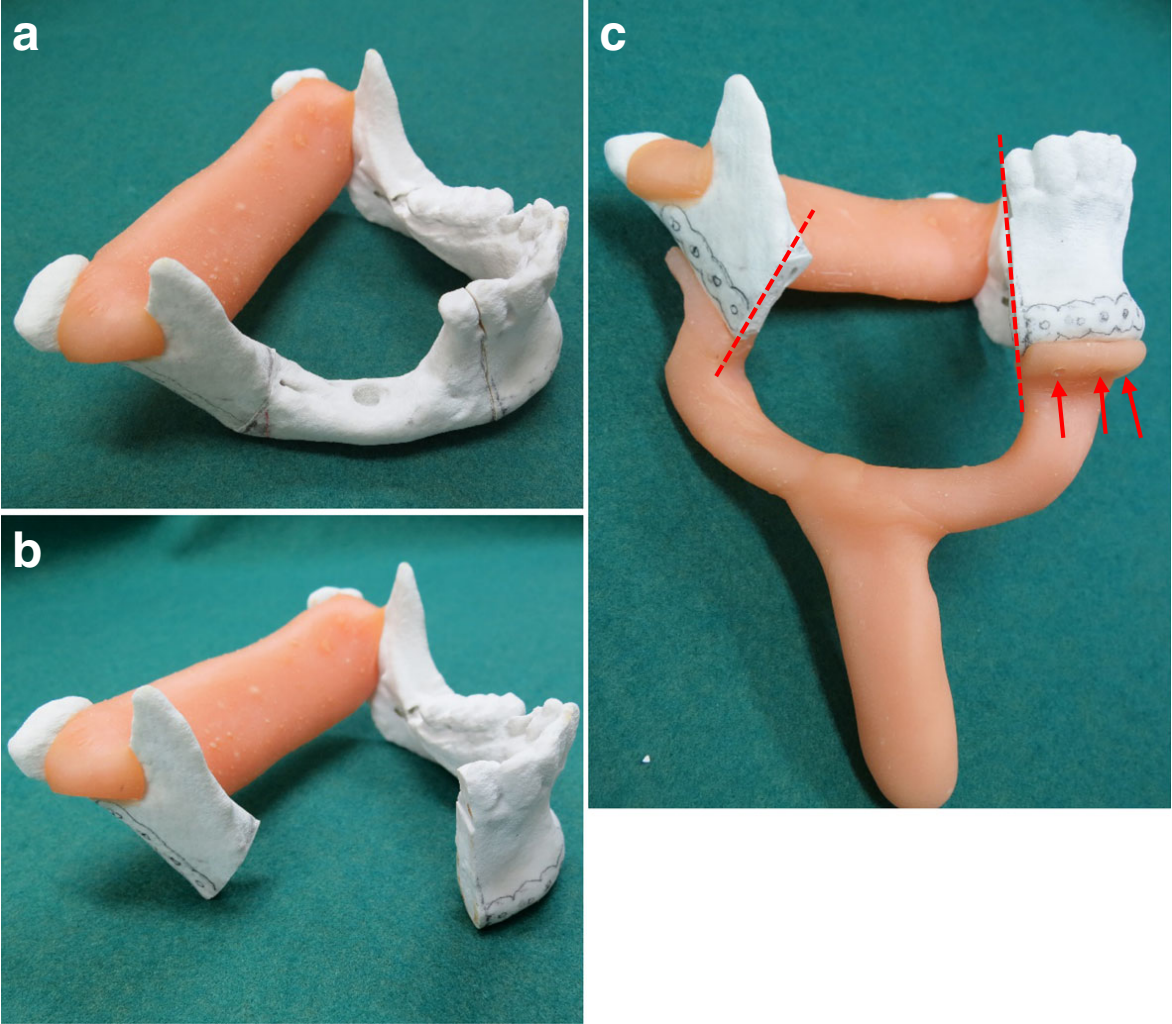
Procedure for making the surgical device in case 2. **a** The *right and left mandibular rami* of the 3D model were connected by an autopolymer resin to keep the preoperative position between proximal and distal segments. **b** Based on the findings in a CT image, the range of resection was determined and segmental resection was performed on the 3D model. The reconstruction titanium plate was bent, and the positions of the plate and holes were marked on the 3D model. **c** The device was made to fit the lower border of the anterior mandible and the posterior border of the mandibular ramus. The surgical device to guide the direction of mandibular resection (*dotted line*) had a U-shaped handle so that adaptation of the device would not interfere with the soft tissue to be removed and had holes (*arrow*) to be fixed on the mandible with screws

Surgical simulation and preparation of the surgical device were performed using a 3D model. First, the right and left mandibular rami of the 3D model were connected by the autopolymer resin to keep the preoperative position between the proximal and distal segments (Fig. 6a). Based on the findings in a CT image, segmental resection was performed on the 3D model with a safety margin of at least 10 mm from the tumor (Fig. 6b). Since it was necessary to extensively resect not only the mandibular bone but also the soft tissue surrounding the mandible, it was impossible to provisionally fix the reconstruction plate on the mandible before mandibular resection. Therefore, a surgical device that not only guides the direction of bone cutting but also restores the preoperative condylar head positioning in the temporomandibular fossa was needed. The device was made to fit the lower border of the anterior mandible and the posterior border of the mandibular ramus. The surgical device to guide the direction of mandibular resection had a U-shaped handle so that adaptation of the device would not interfere with the soft tissue to be removed and had holes to be fixed on the mandible with screws (Fig. 6c). A titanium reconstruction plate was bent so as to be inside at the area of resection in the 3D model.

**Fig. 7:**
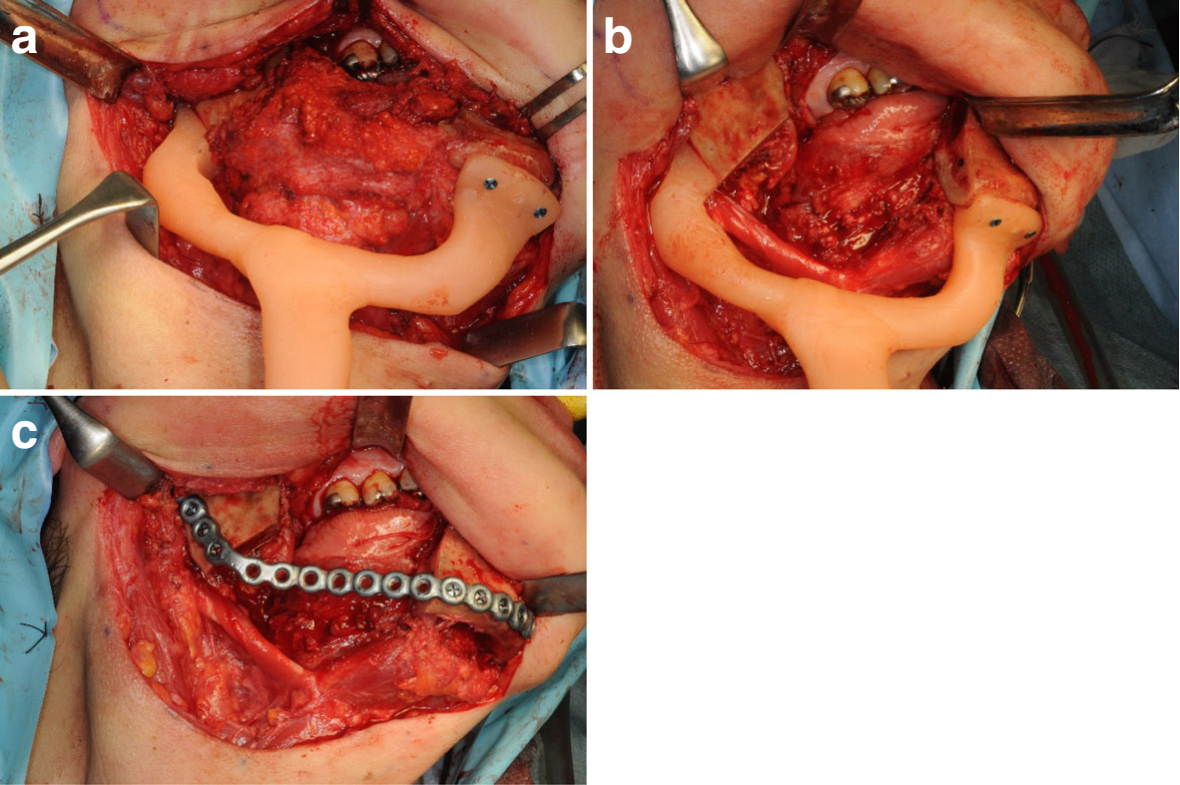
Surgical procedure in case 2. **a** The surgical device was fixed on the mandible with the two screws each at the proximal segment and distal segment. **b** The mandibular resection was performed along the proximal and distal edges of the device fixed to the mandible. **c** After the pre-bent titanium reconstruction plate had been fixed on the proximal and distal segments with screws, the screws used to fix the surgical device were removed

**Fig. 8:**
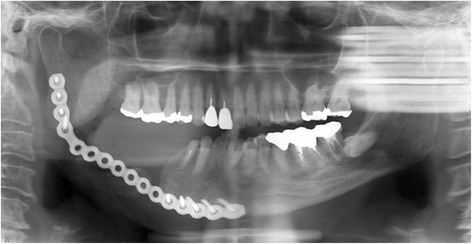
Postoperative panoramic radiograph in case 2. The preoperative relation between the proximal and distal segments and the position of the condylar heads in the temporomandibular fossae were maintained

Segmental mandibular resection was performed via a submandibular approach. The surgical device was fixed on the mandible with two screws each at the proximal segment and distal segment (Fig. 7a). The mandibular resection was performed along the proximal and distal edges of the device fixed to the mandible (Fig. 7b). After the pre-bent titanium reconstruction plate had been fixed on the proximal and distal segments with screws, the screws used to fix the surgical device were removed (Fig. 7c). A panoramic radiograph showed that the mandible had been successfully reconstructed with the reconstruction plate since the preoperative relation between the proximal and distal segments and the position of the condylar heads in the temporomandibular fossae were maintained (Fig. 8).

## Results

We successfully performed the planned accurate segmental mandibulectomy and the precise repositioning of the condylar head by the device in 10 cases.

## Discussion

Previously reported techniques and devices for the restoration of preoperative positioning of the condylar head and the fixation of mandibular segments after segmental resection of the mandible are useful, but they are complex or need considerable time, specialized experience, and dedicated instruments [3, 5–10]. Our technique and device are simple and overcome problems of previously reported techniques. The autopolymer resin for the device is an inexpensive resin used in dental laboratories, and the tools used to make the device are only dental technical carbide and steel bars. The most valuable points of the device are that the preoperative position of the proximal and distal segments of the mandible, even if there are no teeth, can be restored and that the planned mandibular resection can be accurately performed at surgery. Our technique and device are also useful for mandibular reconstruction with a free vascularized osteocutaneous fibula flap. The method described here has been used in 10 cases with no postoperative complication and no deviation of the occlusion due to displacement of the condylar head in the temporomandibular fossa.

## Conclusions

The present technique and device that we developed proved to be simple and useful for restoring the preoperative condylar head positioning in the temporomandibular fossa and the precise resection of the mandible.
